# *N*^6^-Methyladenine DNA Modification in the Woodland Strawberry (*Fragaria vesca*) Genome Reveals a Positive Relationship With Gene Transcription

**DOI:** 10.3389/fgene.2019.01288

**Published:** 2020-01-10

**Authors:** Shang-Qian Xie, Jian-Feng Xing, Xiao-Ming Zhang, Zhao-Yu Liu, Mei-Wei Luan, Jie Zhu, Peng Ling, Chuan-Le Xiao, Xi-Qiang Song, Jun Zheng, Ying Chen

**Affiliations:** ^1^Key Laboratory of Ministry of Education for Genetics and Germplasm Innovation of Tropical Special Trees and Ornamental Plants, Hainan Key Laboratory for Biology of Tropical Ornamental Plant Germplasm, College of Forestry, Natural Rubber Cooperative Innovation Centre of Hainan Province & Ministry of Education of China, Hainan University, Haikou, China; ^2^State Key Laboratory of Ophthalmology, Zhongshan Ophthalmic Center, Sun Yat-sen University, Guangzhou, China; ^3^Institute of Wheat Research, Shanxi Academy of Agricultural Sciences, Linfen, China

**Keywords:** *Fragaria vesca*, DNA 6mA modification, single-molecule real time, gene expression, long non-coding RNA

## Abstract

*N*^6^-methyladenine (6mA) DNA modification has been detected in several eukaryotic organisms, where it plays important roles in gene regulation and epigenetic memory maintenance. However, the genome-wide distribution patterns and potential functions of 6mA DNA modification in woodland strawberry (*Fragaria vesca*) remain largely unknown. Here, we examined the 6mA landscape in the *F. vesca* genome by adopting single-molecule real-time sequencing technology and found that 6mA modification sites were broadly distributed across the woodland strawberry genome. The pattern of 6mA distribution in the long non-coding RNA was significantly different from that in protein-coding genes. The 6mA modification influenced the gene transcription and was positively associated with gene expression, which was validated by computational and experimental analyses. Our study provides new insights into the DNA methylation in *F. vesca*.

## Introduction

DNA methylation, a crucial feature of epigenetic modification, occurs *via* the addition of a methyl group (CH_3_) to DNA nucleotides and plays important roles in regulating genomic imprinting, transposon suppression, gene expression, epigenetic memory maintenance, embryonic development, and tumorigenesis (Bergman and Cedar, 2013; Smith and Meissner, 2013; von Meyenn et al., 2016). 5-Methylcytosine (5mC) and *N*^6^-methyladenine (6mA) are the most common types of DNA methylation. 5mC modification, which involves methylation at the fifth position of the pyrimidine ring of cytosine, is the most extensively studied type of DNA methylation in eukaryotes (Law and Jacobsen, 2010; Jones, 2012). In contrast, 6mA modification, involving methylation at the sixth position of the purine ring of adenine in DNA molecule, is widely present in prokaryotes, where it influences gene expression, DNA replication and repair, cell cycle progression, and host–pathogen interactions (Messer and Noyer-Weidner, 1988; Lu et al., 1994; Collier et al., 2007). However, 6mA DNA modification has also been detected and confirmed recently in plants and animals (Ratel et al., 2006; Luo et al., 2015b), including mammalian DNA (Smith and Meissner, 2013), using current analytical methods.

Several strategies have been employed to identify 6mA DNA modification, including liquid chromatography coupled with tandem mass spectrometry (LC-MS/MS), 6mA immunoprecipitation sequencing (6mA-IPseq), restriction enzyme-based 6mA sequencing (6mA-REseq), and single-molecule real-time (SMRT) sequencing technology. LC-MS/MS facilitates unambiguous detection and quantification of methylated nucleotides (Frelon et al., 2000) and enables high-sensitivity detection of certain low-abundance nucleotide modifications, but cannot identify the genomic locations of 6mA. Although both 6mA-IPseq and 6mA-REseq, which are based on high-throughput sequencing, can locate 6mA sites, their sensitivity of quantification and species specificity in 6mA detection remain to be improved (Roberts and Macelis, 2001; Laird, 2010; Luo et al., 2015a). A recently developed SMRT sequencing, which is based on variances in interpulse duration (IPD) between two successive base incorporations in modified sites of DNA template (Flusberg et al., 2010), is a powerful technique for detection of 6mA modifications at single-nucleotide resolution and single-molecule level. SMRT technology has provided insights into the presence of 6mA in eukaryotes and revealed that 6mA modification is widely present in many eukaryotes, including *Caenorhabditis elegans* (Greer et al., 2015), fungi (Mondo et al., 2017), *Saccharomyces cerevisiae* (Liang et al., 2018b), *Arabidopsis thaliana* (Liang et al., 2018a), *Mus musculus* (Wu et al., 2016), *Oryza sativa* (Zhang et al., 2018; Zhou et al., 2018), and Homo sapiens (Xiao et al., 2017; Xiao et al., 2018). Furthermore, this technology helped to describe features of 6mA related to biological processes (Zhang et al., 2003; Fu et al., 2015; Greer et al., 2015; Liu et al., 2016; Wu et al., 2016; Mondo et al., 2017; Liang et al., 2018a; Liang et al., 2018b; Xiao et al., 2018; Ma et al., 2019). For example, the periodic distribution patterns of 6mA that have been described on a genome-wide scale, within gene regions, and at transcription start sites (TSS) are shown to be indicative of actively expressed genes (Liang et al., 2018a; Xiao et al., 2018).

The Rosaceae is a large plant family containing numerous species of horticultural importance that produce a diverse range of edible fleshy fruits, including apple, cherry, peach, pear, plum, and strawberry (Jung et al., 2008). Among these, the woodland strawberry (*Fragaria vesca*) is an important model system for genetic studies of the Rosaceae family because of its small stature, short generation time, and efficient genetic transformation (Liston et al., 2014; Edger et al., 2018). Recently, it has been speculated that DNA methylation plays a regulatory role in the turning stage of the fleshy fruits of *F. vesca* and in its responses to environmental alteration, and the gene expression of DNA methyltransferases and demethylases was altered when plants are exposed to various abiotic stresses (Gu et al., 2016; Cheng et al., 2018). However, the genome-wide distribution patterns and potential functions of 6mA DNA modification in *F. vesca* still remain largely unknown.

The aims of this study were to 1) reveal the global distribution patterns of 6mA DNA modification in *F. vesca* using the released dataset obtained by PacBio SMRT sequencing technology; 2) describe in detail the level of 6mA modification in the long non-coding RNA (lncRNA) and compare it with that in protein-coding genes; and 3) explore the function of genes with highly methylated DNA by using gene ontology (GO) analysis. The results indicated that 6mA modification is broadly distributed across the woodland strawberry genome, ADSYA, RAGGY, and VACCBA are the most prevalent motifs at 6mA modification sites, and the 6mA density of protein-coding (CDS) regions was significantly enriched and associated with actively expressed genes. The detection and genome-wide distribution profiling of 6mA in *F. vesca* reported in this study provide new insights into DNA methylation in the family Rosaceae.

## Materials and Methods

### Identification of 6mA in the *F. vesca* Genome

The raw data files (accession number SRP125884) of SMRT sequencing reads in h5 format were downloaded from the NCBI Sequence Read Archive (SRA) database (Edger et al., 2018) (**Supplementary Table S1**). The PacBio SMRT analysis platform (version 2.3.0) (http://www.pacb.com/products-and-services/analytical-software/smrt-analysis/analysis-applications/epigenetics) was used to detect 6mA DNA modifications in the *F. vesca* genome. The details of the analysis workflow are as follows. The raw reads were initially aligned to the corresponding reference genome using pbalign with the parameters ‘–seed = 1 –minAccuracy = 0.75 –minLength = 50 –concordant –algorithmOptions = “-useQuality” –algorithmOptions = ‘-minMatch 12 -bestn 10 -minPctIdentity 70.0’’. Thereafter, the polymerase kinetics information was loaded following alignment by loadChemistry.py, and loadPulses scripts of the raw h5 format files were aligned with parameters ‘-metrics DeletionQV, IPD, InsertionQV, PulseWidth, QualityValue, MergeQV, SubstitutionQV, DeletionTag’. The post-aligned datasets were sorted using cmph5tools. The 6mA modification was identified by using ipdSummary.py script with parameters ‘–methylFraction –identify 6mA –numWorkers 4’; 6mA sites with less than 25-fold coverage per chromosome were excluded from further analysis. The reference genome FraVesHawaii_1.0 obtained from NCBI was used to align sequence reads and for gene annotations (Shulaev et al., 2011).

### Bioinformatics Analysis

Genome-wide 6mA DNA modification profiles across all chromosomes of *F. vesca* were obtained using Circos (Krzywinski et al., 2009). The genome was divided into protein-coding gene, non-coding RNA gene, and intergenic regions; the protein-coding gene region was further subdivided into 5′ untranslated region (UTR), CDS, 3′UTR, and intronic regions; these genome features were extracted based on an annotated gff file (FraVesHawaii_1.0) and the R package GenomicFeatures (Lawrence et al., 2013). For each 6mA modification site, we extracted 4 bp upstream and downstream from the 6mA site as described in the literature (Liang et al., 2018a), and then calculated the frequency of each base (A, T, C, and G) and predicted the corresponding motif sequence. Conserved motifs in the flanking regions of 6mA sites were detected using MEME (Bailey et al., 2009) with parameters ‘meme-chip -norc -meme-mod anr -meme-minw 5 -meme-maxw 7 -meme-nmotifs 10 -spamo-skip -fimo-skip’. The corresponding *E* values of the motifs were calculated using Fisher’s exact test. For the profiles of 6mA in genomic features, the genome-wide methylation rate of adenine sites was determined by calculating the mean of 6mA sites relative to all adenine sites. The expected number of methylation sites in each category was calculated by multiplying the total number of adenines in that category by the genome-wide methylation rate. The significant difference of the observed and expected number of methylation sites was determined using the Wilcoxon test.

### Computational Analysis of 6mA Density and Gene Expression

The short-read RNA-Seq raw data of *F. vesca* leaves (cultivar Hawaii-4) were acquired from NCBI (accession number SRR6320486; **Supplementary Table S2**). The RNA-Seq reads were aligned to the FraVesHawaii_1.0 reference genome using TopHat2 with ‘-g 20 -N 2 -p 20 –no-coverage-search’ option (Kim et al., 2013). The values of fragments per kilobase of transcript per million mapped reads (FPKM), calculated by Cufflinks (Trapnell et al., 2010), were used to quantify gene expression. The relationship between gene expression and 6mA density was established according to the same gene symbol from the annotated *F. vesca* genome; all the genes with FPKM value and 6mA density larger than zero were selected for analysis. Within those genes, 6mA density was compared between high-expression genes (*n* = 1,852, FPKM value > average) and low-expression genes (*n* = 10,873, FPKM value < average). Similarly, FPKM was compared between genes with high 6mA density (*n* = 4,949, 6mA density value > average) and low 6mA density (*n* = 7,776, 6mA density values < average). Significant differences in both comparisons were assessed by Student’s *t* test.

### RNA Extraction and Experimental Analysis of Gene Expression

Total RNAs were extracted from young leaves of *F. vesca* using TIANGENRNA Plant Plus Reagent (Tiangen, Beijing, China). cDNA was synthesized using the SuperScriptII system (Invitrogen, Madison, WI, USA) according to the manufacturer’s instructions and diluted eight times for subsequent quantitative real-time PCR (qRT-PCR). Thirty genes were randomly selected from each of the genes with the 100 highest and the genes with the 100 lowest 6mA densities. The selected 60 genes were subjected to qRT-PCR analysis to profile their gene expression using primers listed in **Supplementary Table S3**. qRT-PCR was carried out with SYBR Premix Ex-Taq (Takara, Dalian, China) in a 7500 Real-Time PCR system (Applied Biosystems, Foster City, CA, USA). RT-PCR was performed in reaction volumes of 20 μl, each containing 2 μl of cDNA, 1 μl of 2 mM gene-specific primers, 0.4 μl of ROX Reference Dye (50×), and 10 μl of 2× SYBR Premix Ex-Taq. The relative expression of each gene was presented as a fold change calculated according to the 2^–ΔCt^ method (Livak and Schmittgen, 2001; Schmittgen and Livak, 2008). The glyceraldehyde-3-phosphate dehydrogenase gene was used as an endogenous reference for real-time PCR, and all analyses were performed with three technical and three biological replicates.

### 6mA Distribution Around TSS in lncRNA and Protein-Coding Genes

All 23,106 protein-coding genes and 1,911 lncRNA with their TSS were extracted according to the genome annotation gff file (FraVesHawaii_1.0). To evaluate and compare 6mA distribution pattern between protein-coding genes and lncRNA, we plotted the 6mA occupancy around TSS. The 6mA occupancy represents the relative number of methylated genes against the total number of protein-coding or lncRNA genes in each 50-bp window plotted within a 1-kb region upstream and downstream of the TSS.

### GO Enrichment Analysis of High-6mA-Density Genes

All protein-coding genes were classified by the parameter *K* = log_2_(FC) into genes with high 6mA density (*K* > 1, *n* = 993), low 6mA density (*K* < −1, *n* = 4,080), moderate 6mA density (|*K*| ≤ 1, *n* = 10,280), and non-6mA genes (*n* = 7,753); FC is the fold change between particular 6mA density of a gene and the average value. The above classes of methylated protein-coding genes and protein-coding genes without methylations were subjected to GO analysis. Due to the lack of GO terms for *F. vesca*, we firstly performed GO annotation by using blast2go (Conesa and Gotz, 2008). The gene sequences were aligned against NCBI non-redundant (nr) based on blastx with a cutoff *E* value = 1E−5, then the results were used for GO term mapping and submitted to agriGO for GO classification (Tian et al., 2017). The false discovery rate-adjusted *P* value <0.01 was considered statistically significant.

### 6mA Identification in Latest Genome Version

The reference genome that we used in this study is the mainstream version from NCBI with a good annotation. To assess the reliability of this study, we re-aligned detected 6mA loci sites with the latest reference genome v4.0 (Edger et al., 2018). The latest genome of *F. vesca* can be found in GDR (genome database for Rosaceae) database (https://www.rosaceae.org/analysis/252). According to the annotation of v4.0 genome, we divided the protein-coding genes regions into 5′UTR, CDS, intron, and 3′UTR. However, we mapped published lncRNA sequences (Kang and Liu, 2015) to v4 genome to identify the lncRNA in re-align reference because of lack of ncRNA information in v4.0 genome annotation. Besides, given the v4.0 genome size is 20M larger than that in v1.0, the lower coverage cutoff (15×) of 6mA was used to filter the low cover 6mA in genome v4.0.

## Results

### Overview of 6mA Modifications in the *F. vesca* Genome

**Figure 1:**
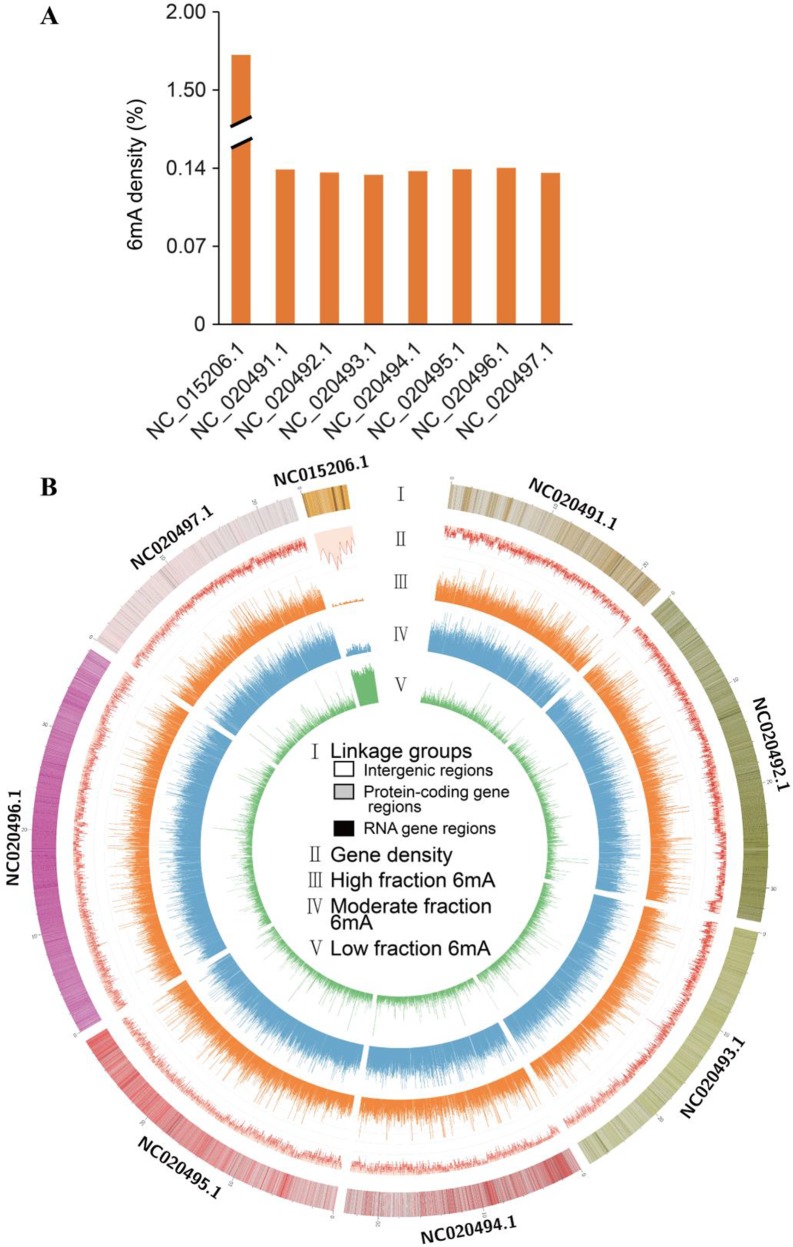
Distribution of *N*^6^-methyladenine (*6mA*) in *Fragaria vesca* v1.0 genome. **(A)** Density of 6mA modification of *F. vesca* linkage groups. **(B)** Circos plot of 6mA in the *F. vesca* genome. *I*: The seven linkage groups and chloroplast complete genome (NC_015206.1); *light color* represents intergenic regions; *ashen color* represents protein-coding gene regions; *dark color* represents RNA gene regions. *II*: Gene density. *III*: High 6mA methylation (65–100%). *IV*: Moderate 6mA methylation (35–65%). *V*: Sparse 6mA methylation (0–35%).

A total of 160,256 DNA 6mA sites were detected in reference genome v1.0 from NCBI, thereby confirming the presence of 6mA modifications in Rosaceae (**Table 1** and **Supplementary_Inf01**). The 6mA density (6mA/A) represented approximately 0.139% of the total adenines in the genomic DNA (**Table 1**), and it was lower than that previously detected in the fungus *Hesseltinella vesiculosa* (2.8%) (Mondo et al., 2017), *C. elegans* (~0.7%) (Greer et al., 2015), and *Chlamydomonas reinhardtii* (~0.4%) (Fu et al., 2015), but higher than that detected in yeast (0.013%) (Liang et al., 2018b), *Drosophila* (0.07%) (Zhang et al., 2015), *H. sapiens* (0.051%) (Xiao et al., 2018), and *A. thaliana* (0.048%) (Liang et al., 2018a). The 6mA sites were broadly distributed across both the linkage groups and the chloroplast genome of *F. vesca*. Among all linkage groups, the third linkage group (NC_020493.1) and the chloroplast genome had the lowest (0.134%) and highest (1.725%) 6mA densities, respectively (**Table 1** and **Figure 1A**).

**Table 1:** Density of *N*^6^-methyladenine (6mA) across the *Fragaria vesca* genomic DNA.

Linkage groups	Size	No. A bases(−)	No. A bases(+)	No. A bases	No. 6mA sites(−)	No. 6mA sites(+)	No. 6mA sites	Density(−)	Density(+)	Density
NC_020491.1	22,681,039	6,567,320	6,591,021	13,158,341	8,952	9,305	18,257	0.141%	0.136%	0.139%
NC_020492.1	33,308,843	9,638,098	9,648,140	19,286,238	13,054	13,204	26,258	0.137%	0.135%	0.136%
NC_020493.1	27,879,571	8,167,868	8,116,646	16,284,514	11,122	10,705	21,827	0.132%	0.136%	0.134%
NC_020494.1	23,292,877	6,727,705	6,722,413	13,450,118	9,208	9,264	18,472	0.138%	0.137%	0.137%
NC_020495.1	29,328,693	8,565,031	8,576,933	17,141,964	11,920	11,893	23,813	0.139%	0.139%	0.139%
NC_020496.1	38,222,195	11,129,178	11,162,536	22,291,714	15,731	15,536	31,267	0.139%	0.141%	0.140%
NC_020497.1	23,403,891	6,867,841	6,891,017	13,758,858	9,155	9,521	18,676	0.138%	0.133%	0.136%
NC_015206.1*	155,691	49,354	48,400	97,754	883	803	1,686	1.659%	1.789%	1.725%
Total	198,272,800	57,712,395	57,757,106	115,469,501	80,025	80,231	160,256	0.139%	0.139%	0.139%

*NC_015206.1 represents complete genome of chloroplast.

The methylation levels at each 6mA site across the genomic DNA were profiled and grouped into the following three fractions: low (0–35%), moderate (35–65%), and high (65–100%) (**Figure 1B**). All three levels of methylation were prevalently distributed across seven linkage groups and the chloroplast genome. High levels were detected in seven linkage groups (**Figure 1B**), indicating that most adenine bases at 6mA sites were modified. Moreover, the level of 6mA modification in the chloroplast genome was substantially different from that in the seven linkage groups, where gene density level was high compared with others (**Figure 1B**).

### 6mA Consensus Motifs in *F. vesca*

**Figure 2:**
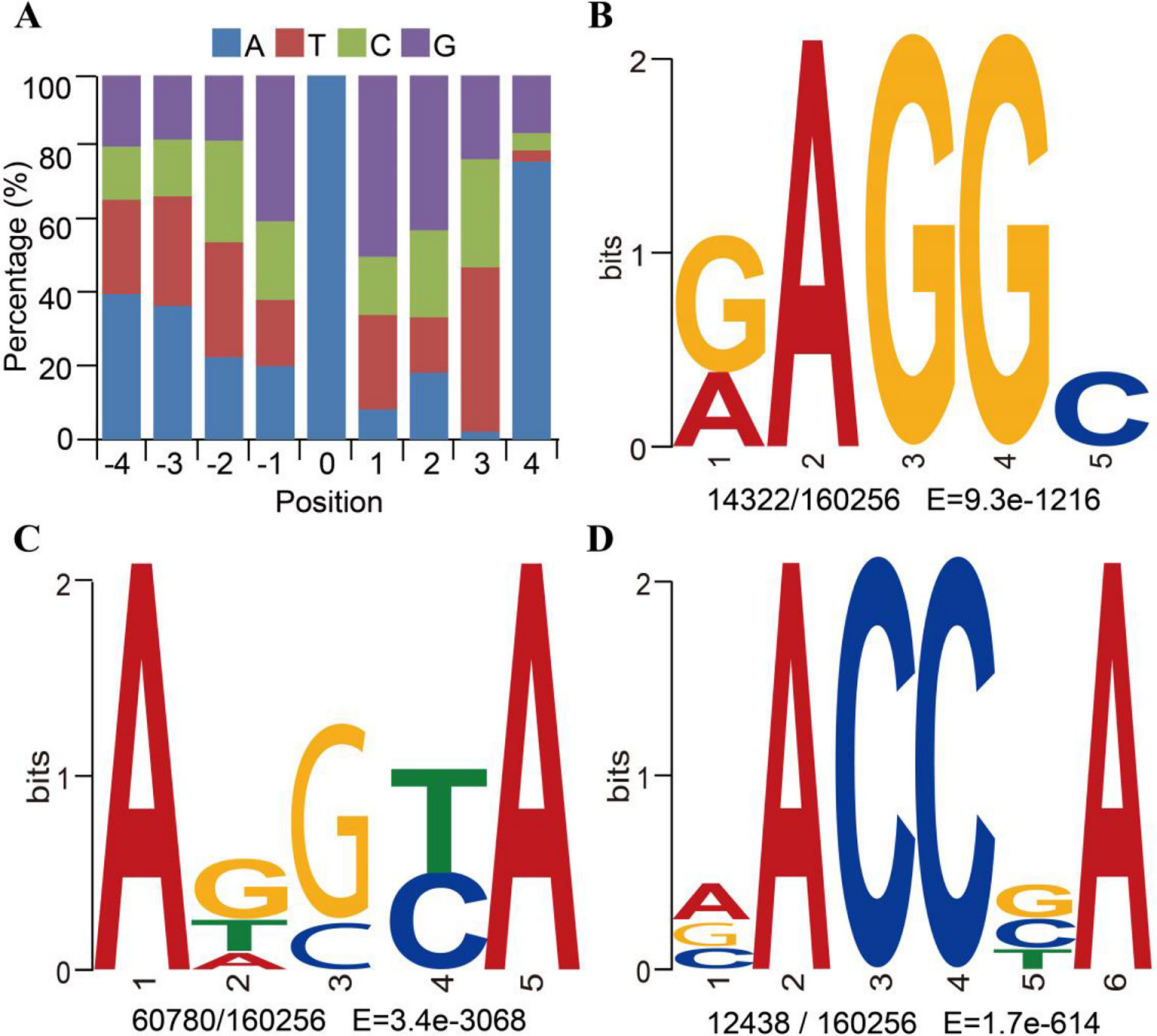
Motif sequences of *N*^6^-methyladenine (*6mA*) modification sites in *Fragaria vesca* v1.0 genome. **(A)** Content percentage of the bases A, T, C, and G in the upstream and downstream 4 bp of 6mA sites (position “0”). **(B**–**D)** Motif sequences of 6mA detected by MEME assay: RAGGY **(B)**; ADSYA **(C)**; and VACCBA **(D)**. Sequence logo representations of the consensus motifs containing 6mA sites identiﬁed by MEME. The number of occurrences of each motif relative to the total number of 6mA-containing motifs and the corresponding *E* value generated by MEME are *shown under the logo*.

To determine whether the identified 6mA sites in *F. vesca* shared consensus sequence elements, we further investigated the identity of the bases in the sequences flanking the 6mA sites and performed a search for significant enrichment of consensus motifs using MEME (Bailey et al., 2009). There was a high probability (frequency higher than 40%) of guanine (G) at positions 1 bp upstream and downstream from the 6mA modification sites (**Figure 2A** and **Supplementary_Inf01**). Three prominent motif sequences were also detected at 6mA sites in the *F. vesca* genome. The RAGGY motif was signiﬁcantly enriched (**Figure 2B**), which was consistent with the common AGG 6mA motif sequence identified in *C. elegans* (Greer et al., 2015), *A. thaliana* (Liang et al., 2018a), and *H. sapiens* (Xiao et al., 2018). The frequency of adenine (A) in the 4-bp sequence downstream of the 6mA modification sites was as high as 76% (**Figure 2A**), and enrichment of adenine was evident in the motif sequence patterns ADSYA and VACCBA, which are similar to the ANYGA and ACCT motifs, respectively, detected in *A. thaliana* (Liang et al., 2018a) (**Figures 2C** and **D**). The detection of these motifs in the *F. vesca* genome can thus be considered reliable evidence of the occurrence of 6mA modification in this plant.

### Genomic Distribution of 6mA Modifications

**Figure 3:**
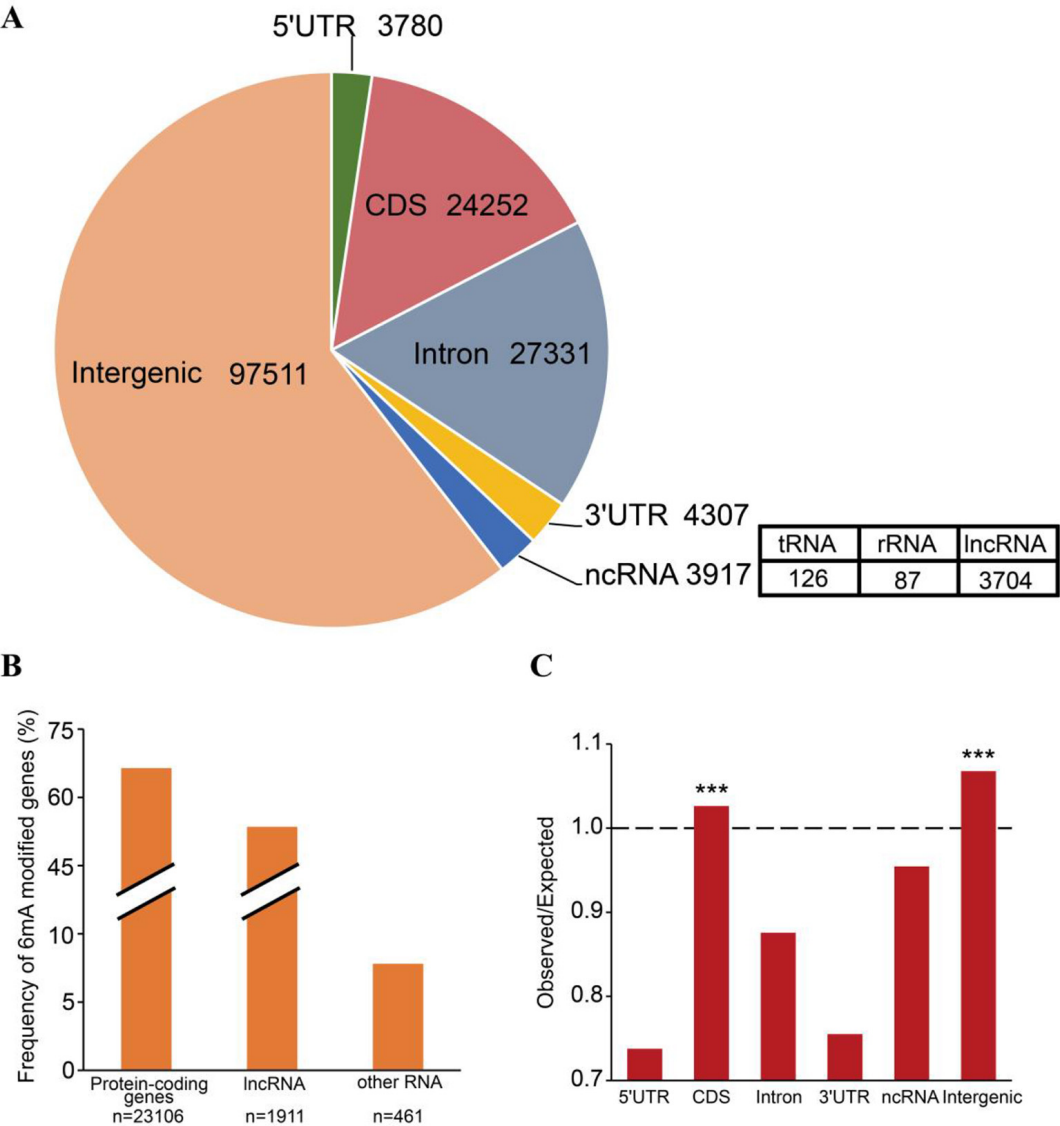
Distribution of *N*^6^-methyladenine (*6mA*) in genomic features of *Fragaria vesca* v1.0 genome. **(A)** Number of 6mA modification sites distributed in 5′UTR, coding sequence (*CDS*), 3′UTR, and intronic regions of protein-coding genes, non-coding (nc)RNA, and intergenic regions. ncRNA included tRNA, rRNA, and lncRNA. **(B)** The frequency of 6mA-methylated genes in protein-coding RNA, lncRNA, and other RNA that included tRNA and rRNA. **(C)** Comparison of observed versus expected distributions of 6mA modifications in each functional element. The expected distribution of methylation sites was calculated by the total number of adenines in each category multiplied by the genome-wide 6mA density. The significant difference between the observed and expected numbers of methylation sites was determined using the binomial test, ***p < 0.001.

6mA enrichment in specific genomic features is associated with the functions of 6mA DNA methylation (Jones, 2012; Smith and Meissner, 2013). We investigated the distributions of 6mA in different functional regions, namely the non-coding RNA gene, intergenic, and protein-coding gene regions. The protein-coding genes were further subdivided into the 5′UTR, CDS, 3′UTR, and intronic regions (**Supplementary_Inf02**). The results indicated that most of the 6mA modification sites were located within intergenic regions of the genome (**Figure 3A**). For all annotated genes in the genomic DNA, we found approximately 16,412 genes (64.42%) harboring 6mA modification sites in 5′UTR, CDS, 3′UTR, and intronic regions (**Figure 3B** and **Supplementary_Inf02**). Surprisingly, 6mA modification density was significantly enriched in the CDS region of methylated genes (*p* < 0.001, binomial test; **Figure 3C**), but not in the 5′UTR, intronic and 3′UTR regions, suggesting that 6mA modification is correlated with gene function.

### Positive Relationship of 6mA Modification With Gene Transcription

**Figure 4:**
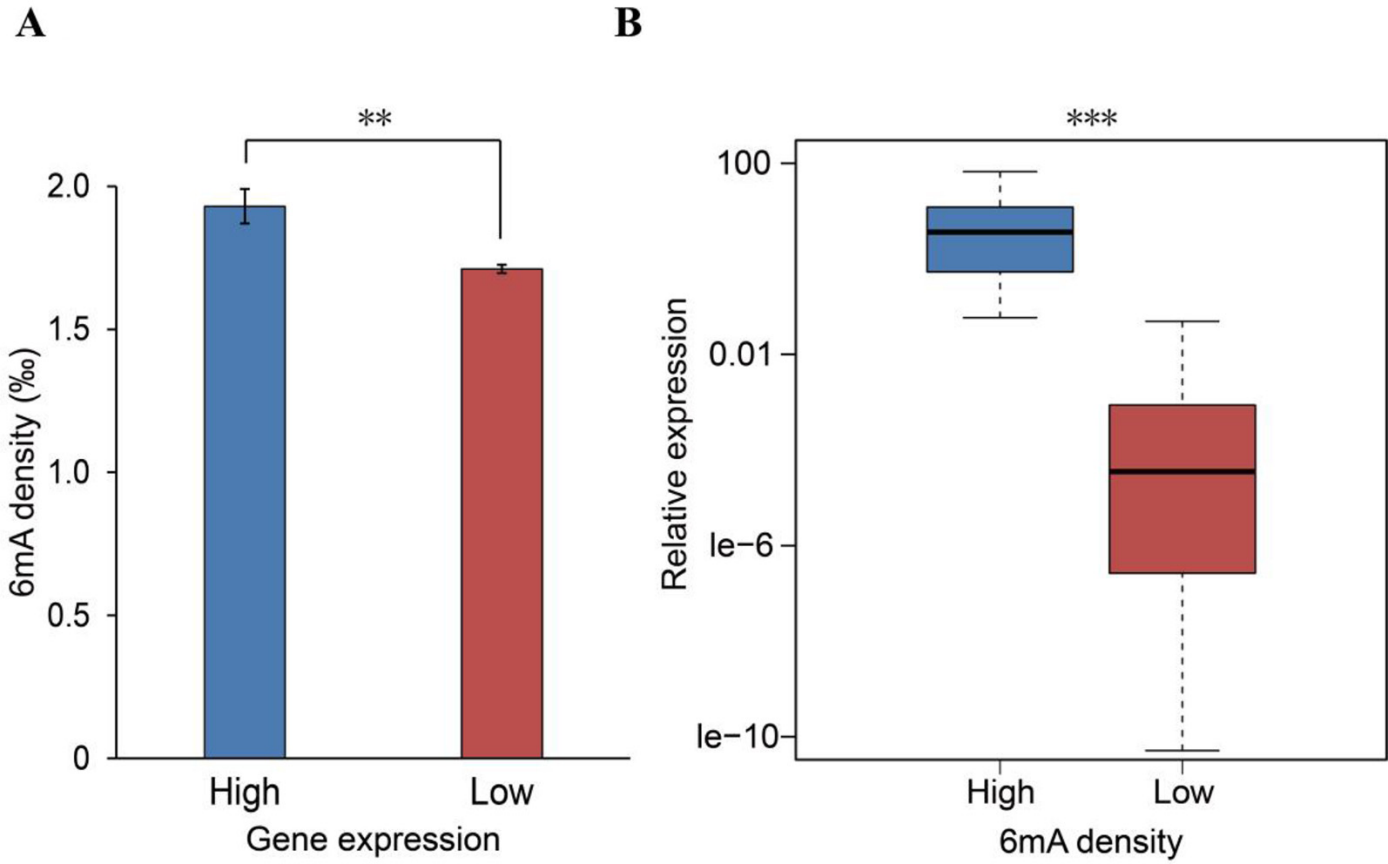
Positive association between *N*^6^-methyladenine (*6mA*) density and gene expression. **(A)** Computational analysis of the 6mA density in low-expression (*n* = 10,873) and high-expression (*n* = 1,852) genes (mean ± SEM, **p < 0.01). **(B)** Experimental analysis of 60 randomly selected genes from among those with the highest (30) and lowest (30) 6mA methylation densities (***p < 0.001). *P* values were calculated by Student’s *t* test.

6mA DNA methylation has recently been reported to affect gene expression in plants, animals, and humans (Fu et al., 2015; Wu et al., 2016; Liang et al., 2018a; Xiao et al., 2018). Given that the potential role of 6mA affecting gene expression in Rosaceae remains elusive, we investigated the influences of the 6mA modification on gene expression. Initially, we obtained RNA-Seq data for *F. vesca* leaves (cultivar Hawaii-4) from NCBI (**Supplementary Table S2**) and compared the 6mA density between the high- and low-expression genes. We found that genes with higher RNA expression have a significantly higher density of 6mA (*p* < 0.05, Student’s *t* test; **Figure 4A** and **Supplementary_Inf03**). Besides, the comparison of the expression between the high-6mA-methylation genes and low-6mA-methylation genes produced similar results (**Supplementary Figure S1**). The positive association between 6mA density and transcription was further verified by profiling the RNA expression of 60 genes randomly selected from genes with the highest and genes with the lowest 6mA density (**Supplementary Table S3**). The results showed that the gene expression of the high-methylation group was significantly higher than that of the low-methylation genes (*p* < 0.001, Student’s *t* test; **Figure 4B**). These findings thereby indicate that 6mA DNA modification may be a marker for actively transcribed genes in *F. vesca*.

### Comparison of 6mA Pattern in Protein-Coding Genes and lncRNA

**Figure 5:**
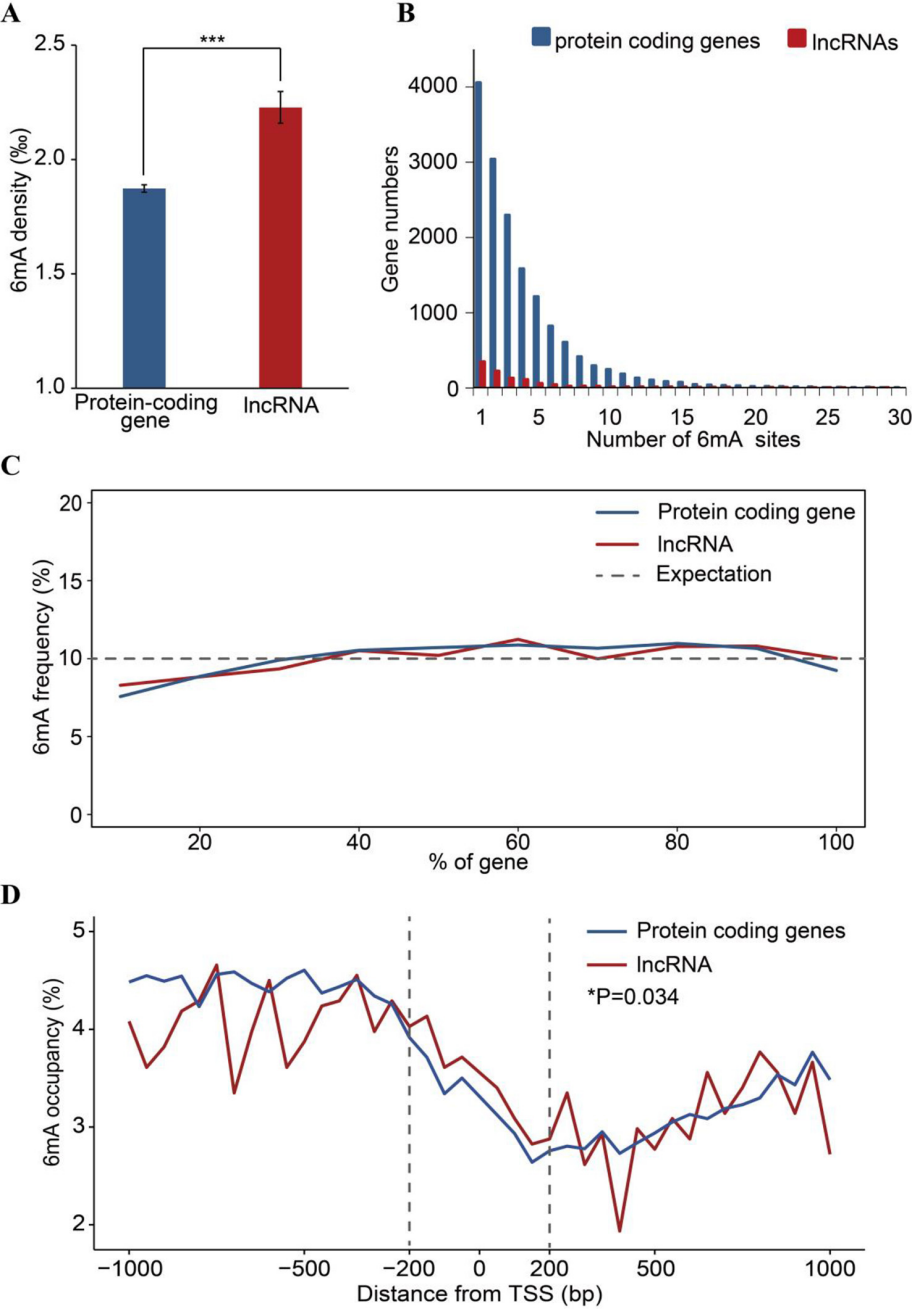
Comparison of 6mA methylation in long non-coding (lnc)RNA and protein-coding genes. **(A)** Different 6mA modification densities in lncRNA and protein-coding genes (***p < 0.001, Student’s *t* test). **(B)** Statistics of gene numbers with different 6mA sites between lncRNA and protein coding genes. **(C)** Frequency of 6mA sites at relative positions in lncRNA and protein-coding genes. **(D)** Distribution of 6mA occupancy around the transcription start site (*TSS*) of lncRNA and protein-coding genes (6mA occupancy represents the relative number of methylated genes against the total number of protein-coding or lncRNA genes in each 50-bp window plotted within a 1-kb region upstream and downstream of the TSS). The 200-bp regions upstream and downstream of the TSS are marked by *dashed lines* (*p < 0.05, Student’s *t* test).

To analyze the 6mA density in coding and noncoding regions, protein-coding and lncRNA genes were examined based on annotation of the *F. vesca* genome. We found that the percentage of lncRNA genes harboring 6mA modification sites (53.53%) was less than that for protein-coding genes (66.45%) (**Figure 3B** and **Supplementary Table S4**), whereas the 6mA density in lncRNA was significantly higher than that in protein-coding genes (*p* < 0.001, Student’s *t* test; **Figure 5A** and **Supplementary_Inf03**). This result indicates that the 6mA in lncRNA may be associated with certain specific functions that may be divergent from the functions in protein-coding genes. Furthermore, the enrichment of 6mA sites in lncRNA and protein-coding genes revealed a similar trend between these genes regarding the number of 6mA sites in each gene, where half of the lncRNA and protein-coding genes were enriched at one to three sites (**Figure 5B** and **Supplementary_Inf04**).

Additionally, the number of 6mA sites at the 5′ ends of lncRNA and protein-coding genes was lower than that in other regions of these genes (**Figure 5C**). Previous studies have reported that the enrichment of 6mA in TSS at the 5′ end of genes activates gene expression (Greer et al., 2015; Liang et al., 2018a; Xiao et al., 2018). Therefore, we further analyzed the distribution pattern of 6mA sites in the TSS of protein-coding genes and lncRNA, which may regulate gene expression in *cis* or in *trans* (Kopp and Mendell, 2018). We determined the number of 6mA-methylated genes in consecutive 50-bp windows throughout the 1-kb regions upstream and downstream of the TSS. The 6mA occupancy, which represents the relative number of protein-coding or lncRNA genes containing 6mA sites against the total number of protein-coding or lncRNA genes, is shown in **Figure 5D** and **Supplementary_Inf05**. Local depletions of 6mA sites detected at the TSS in protein-coding and lncRNA genes were similar to the changes around TSSs observed in previous studies (Fu et al., 2015; Edger et al., 2018; Liang et al., 2018a; Zhang et al., 2018; Zhou et al., 2018). However, the occurrence of 6mA in the 200-bp regions upstream and downstream of the TSS in lncRNA genes was higher than that in the same regions of protein-coding genes (**Figure 5D**). These observations imply that the 6mA pattern differs between lncRNA genes and protein-coding genes.

### High-6mA-Density Genes Are Associated With Photosynthesis

**Figure 6:**
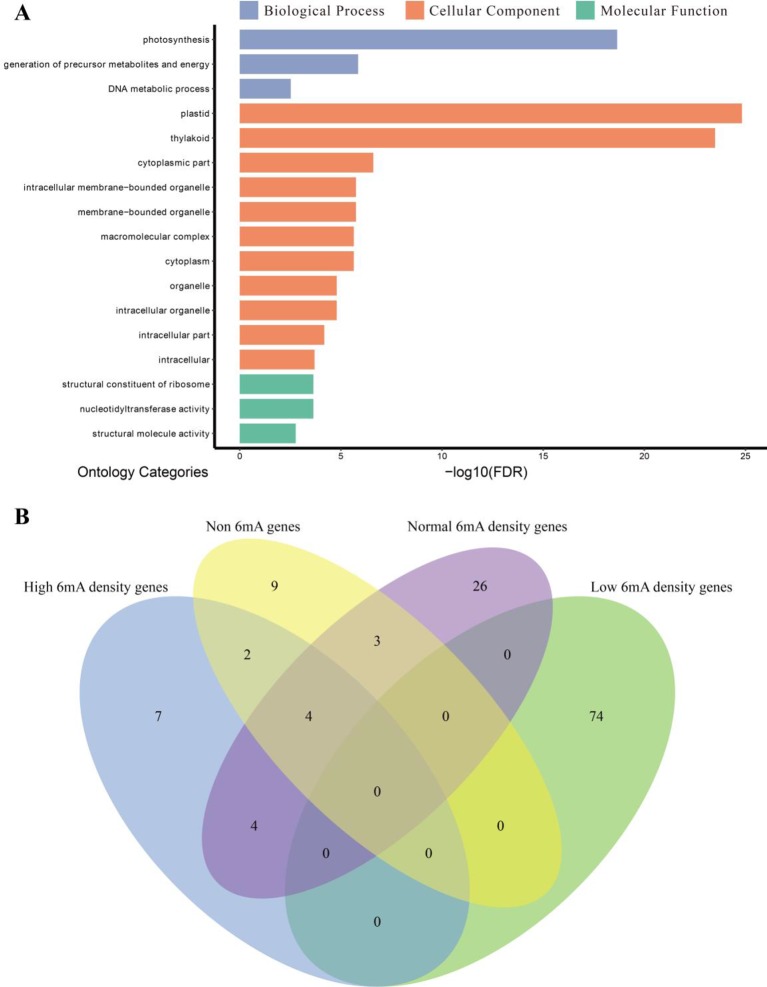
Gene ontology (GO) enrichment analysis. **(A)** GO enrichment categories of high-methylation-density protein-coding genes (*n* = 993) in *Fragaria vesca*. The GO categories are listed with the false discovery rate-adjusted *P* value <0.01. **(B)** Venn diagram of enrichment categories of four gene sets.

To provide insights into the functions of highly 6mA-methylated genes, the methylated protein-coding genes were classified into genes with high, low, and moderate levels of 6mA density. The GO enrichment analysis was performed for each group, and the non-6mA-modified genes were used as a control (**Figure 6A** and **Supplementary Figure S2**). We found that the highly methylated genes were significantly enriched in cellular component (**Figure 6A**), and low-6mA-density genes presented difference compared to the other three groups in GO enrichment analysis (**Figure 6B** and **Supplementary Figure S2**). Firstly, the GO categories enriched by low-6mA-density groups were more than others and with obvious statistical significance (**Supplementary Figure S2** and **Supplementary_Inf06**); secondly, low-6mA-density gene groups enriched in a mass of biological process categories that are more than other groups (**Supplementary Figure S2** and **Supplementary_Inf06**); finally, the GO categories of low 6mA density show strict specificity from the other three gene groups (**Figure 6B**). Given the genomic DNA of sample was isolated from young leaves of dark-treated plant (**Supplementary Table S1**), the special above results of GO enrichment for four gene groups in various 6mA modification levels may be associated with response of 6mA modification to environmental stress.

## Discussion

The recent extensive studies on 6mA DNA modification in eukaryotes have revealed that 6mA plays important roles in gene expression, epigenetic memory maintenance, embryonic development, and tumorigenesis (Greer et al., 2015; Liang et al., 2018a; Xiao et al., 2018). Here, we report on the occurrence of 6mA modification in the horticultural plant *F. vesca* using SMRT sequencing technology, which opens up a new and promising dimension of epigenetic research on the family Rosaceae. Our findings indicate that 6mA sites are broadly distributed across the *F. vesca* genome, particularly in the chloroplast genome, and that the gene coding sequences are the regions harboring the highest density of 6mA sites. We detected three motifs, ADSYA, RAGGY, and VACCBA, characteristic of 6mA modification sites that are broadly consistent with the motifs identified in other eukaryotic organisms (Greer et al., 2015; Liang et al., 2018a; Xiao et al., 2018). Furthermore, we found that 6mA modification is positively associated with gene expression, which was validated by computational and qRT-PCR analysis. Moreover, the distribution pattern of 6mA sites in lncRNA genes was significantly different from that in protein-coding genes. In addition, the GO enrichment analysis for the high 6mA modification density revealed that DNA 6mA methylation in leaf tissue plays an important role in photosynthesis processes.

Rosaceae is one of the most speciose eudicot families and includes a diverse range of important horticultural crops (apples, apricots, blackberries, cherries, peaches, pears, plums, raspberries, roses, and strawberries) (Shulaev et al., 2008). Woodland strawberry (*F. vesca*) represents an important model system for studying various aspects of the Rosaceae family. To date, however, there have been no genome-wide analyses of the epigenetic methylation of DNA related to 6mA modification in Rosaceae. A genome-wide distribution of 6mA in *F. vesca* revealed a high density of 6mA modification sites only on one chromosome (chloroplast). Similar results were reported for *A. thaliana* (Liang et al., 2018a) and *H. sapiens* (Xiao et al., 2018), and have been associated with chromosome size and its relative gene number (Liu et al., 2019). Furthermore, a large number of short conserved sequences, such as the AGG motif, were found at 6mA locations; similar conserved sequences were identified in *C. elegans* (Greer et al., 2015), *A. thaliana* (Liang et al., 2018a), and *H. sapiens* (Xiao et al., 2018). This similarity in distribution patterns and conserved sequences further confirmed the occurrence of 6mA in *F. vesca* and suggested that the generative mechanism for DNA 6mA modifications is shared by different organisms.

The genome-wide distribution patterns and potential functions of 6mA in *F. vesca* were assessed based on the 6mA methylation and RNA expression information obtained from the leaf tissue of *F. vesca*. Computational analysis of the genes was verified experimentally by qRT-PCR. Considering the complexity of spatiotemporal gene expression and the repeatability of biological results, we selected the genes with high and low 6mA density for comparison; most of these genes were constitutive genes in experimental analysis. The consistent results in dynamic epigenome were validated in these two separate analyses and reliably revealed the positive relationship between 6mA density and gene expression (**Figure 4** and **Supplementary Figure S1**). These results were consistent with pervious findings in *H. sapiens* (Xiao et al., 2018), *C. elegans* (Greer et al., 2015), *C. reinhardtii* (Fu et al., 2015), fungi (Mondo et al., 2017), and *O. sativa* (Zhang et al., 2018; Zhou et al., 2018), thereby implying that 6mA DNA modification is associated with the activation of gene expression in *F. vesca*. This supposition should ideally be further confirmed in other species of the family Rosaceae.

According to the reference genome annotation file (FraVesHawaii_1.0), there were 23,106 protein coding genes, 8 rRNA, 453 tRNA, and 1,911 lncRNA. The untranslated lncRNA genes had diverse functions, including transcriptional regulation in *cis* or *trans* and protein regulation (Kopp and Mendell, 2018). To date, 6mA DNA modification has been studied in eukaryotes, and the results of those studies revealed the functional role of 6mA methylation in gene transcription. However, the distribution pattern and potential function of 6mA in lncRNA are still unclear. This study is the first to analyze the distribution patterns of 6mA in lncRNA genes and compare them with the 6mA distribution in protein-coding genes. We found that the percentage of lncRNA genes harboring 6mA modification sites was less than that of protein-coding genes (**Supplementary Table S4**), whereas the density of 6mA sites in lncRNA was significantly higher than that in protein-coding genes (**Figure 5A**), indicating that in lncRNA, 6mA may be involved in certain specific functions that differ from those in protein-coding genes. The functional mechanism of 6mA methylation in lncRNA is currently undetermined and warrants further studies.

The GO analysis revealed that 6mA prefers to enrich in related genes with cellular component in young leaf under dark condition. And the obvious significance of GO categories in low-6mA-density gene groups demonstrated that 6mA may maintain low density in lots of genes related with biological process under specific circumstance. It may be rational, the young seedling encountered no light condition needs to suitably deactivate some genes to maintain basic life active (cellular component) and lower the 6mA density to respond to the specific condition (biological process).

To assess the reliability of our study, we re-aligned the sequencing dataset to the latest reference genome v4.0 from GDR database and detected 6mA loci sites. Due to the latest genome being larger (20M) than genome v1.0, the total detected 6mA sites from the re-aligned reference were 180,910, which was slightly more than that in genome v1.0 (160,256), while the 6mA density in v4.0 (0.135%) is almost consistent with v1.0 (0.139%) (**Tables 1** and **Supplementary Table S5**). Similarity and coincident tendency were demonstrated in the detected 6mA loci sites of over half genome size (**Supplementary Figure S3A**). Besides, we also analyzed the three methylation levels grouped by the same standard across the v4.0 genome and found the same results of the broad distribution of 6mA sites with levels across the whole genome, as well as a high proportion of high fractions and moderate fractions in 6mA (**Supplementary Figure S3B**). Furthermore, we evaluated the features of the bases around 6mA sites in v4.0 genome, and three features that were consistent with v1.0 were also observed: 1) the high probability (frequency higher than 40%) of guanine (G) at positions 1 bp upstream and downstream from the 6mA sites; 2) the low probability (frequency lower than 3%) of adenine (A) at positions 3 bp downstream from the 6mA sites; and 3) the high probability (frequency higher than 60%) of adenine (A) at positions 4 bp downstream from the 6mA sites (**Supplementary Figure S4A**). Besides, three prominent motifs, ANHGA, SNAGGY, ACCGA, were detected in v4.0 (**Supplementary Figures S4B**–**D**), which shared the main characteristic with the motifs detected from v1.0 (ADSYA, RAGGY, and VACCBA) (**Figures 2B**–**D**). Importantly, we reanalyzed the relationship between gene expression and 6mA modification in v4.0 by comparing 6mA density between high-expression genes (*n* = 454) and low-expression genes (*n* = 4429). We found that the positive tendency between 6mA modifications with gene transcription was also in v4.0 (**Supplementary Figure S5**). Overall, the result from v4 genome supports previous findings, and given the v1 genome is the default version in NCBI with a good annotation and more friendly application, we simultaneously used genome v1.0 and v4.0 in this study.

In addition to the detection of 6mA DNA modification, SMRT sequencing technology is a highly efficient approach for resolving other important epigenetic DNA modifications at single-nucleotide resolution (Eid et al., 2009; Smith and Meissner, 2013). For example, *N*^4^-methylcytosine (4mC) may have a function in epigenetic regulation that is complementary to that of 6mA (Liu et al., 2019). This type of DNA methylation also deserves further investigation.

## Data Availability Statement

The PacBio datasets of the *F. vesca* analyzed for this study can be found in Sequence Read Archive (SRA) of NCBI https://www.ncbi.nlm.nih.gov/sra/?term = SRP125884. Short-read RNA sequencing data analyzed for this study can be found in SRA https://www.ncbi.nlm.nih.gov/sra/?term\ = SRR6320486. The assembly and annotation files of the *F. vesca* reference v1.0 genome analyzed for this study can be found in NCBI https://www.ncbi.nlm.nih.gov/genome/3314. The *F. vesca* V4 assembly and annotation are made publicly available on the Genome Database for Rosaceae (https://www.rosaceae.org/species/fragaria_vesca/genome_v4.0.a1) and the GigaScience database (http://gigadb.org/dataset/100372). The 6mA data generated for this study is included in **Data Sheet 2**.

## Author Contributions

S-QX and YC conceived the project and designed the experiments. J-FX and X-MZ detected 6mA and performed the bioinformatics analysis. J-FX, Z-YL, M-WL, JiZ, and PL collected and preprocessed the SMRT sequencing data. JuZ performed the experiments for the 6mA-associated gene expression. S-QX, J-FX, and JiZ wrote the paper. X-QS and C-LX revised the paper.

## Funding

This work was supported by the National Natural Science Foundation of China (31760316, 31600667, 31701146, 31871326), Priming Scientific Research Foundation of Hainan University (KYQD(ZR)1721), and Shanxi Province Research Program (201803D421021). Natural Science Foundation of Jiangsu Province (BK20160582). The funders had no role in study design, data collection and analysis, decision to publish, or preparation of the manuscript.

## Conflict of Interest

The authors declare that the research was conducted in the absence of any commercial or financial relationships that could be construed as a potential conflict of interest.

## Abbreviations

6mA, *N*^6^-methyladenine DNA modification; 5mC, 5-Methylcytosine; SMRT, single-molecule real-time; lncRNA, long non-coding RNA; GO, gene ontology; LC-MS/MS, liquid chromatography coupled with tandem mass spectrometry; 6mA-Ipseq, 6mA immunoprecipitation sequencing; 6mA-Reseq, restriction enzyme-based 6mA sequencing; IPD, interpulse duration; TSS, transcription start sites; UTR, untranslated region; CDS, coding sequence; qRT-PCR, quantitative real-time polymerase chain reaction; FPKM, fragments per kilobase of transcript per million mapped reads; SRA, Sequence Read Archive.

## Supplementary Material

Data Sheet 1 Includes Supplementary Tables 1-5 and Supplementary Figures 1-5.

Data Sheet 2 Supplementary_Inf01: Information about the total identified 6mA modifications aligned with v1.0 and v4.0 genome.

Data Sheet 3 Supplementary_Inf02: 6mA density in genomic features.

Data Sheet 4 Supplementary_Inf03: The levels of 6mA methylation and expression of all genes from v1.0 and v4.0 genome.

Data Sheet 5 Supplementary_Inf04: 6mA site distribution in protein-coding genes and long noncoding RNA from v1.0 genome.

Data Sheet 6 Supplementary_Inf05: The 6mA number and 6mA occupancy in the regions 1 kb upstream and downstream from the transcription start sites (TSS) of the protein coding genes and long non-coding RNA (lncRNA) from v1.0 genome.

Data Sheet 7 Supplementary_Inf06: Gene ontology function and enrichment category.
