# Supplementary material for: N6-Methyladenine DNA Modification in the Woodland Strawberry (Fragaria vesca) Genome Reveals a Positive Relationship With Gene Transcription
Source: Front Genet. 2020 Jan 10;10:1288. doi: 10.3389/fgene.2019.01288 (PMC6967393; doi:10.3389/fgene.2019.01288)
Supplement: Data Sheet 1 — Includes Supplementary Tables 1-5 and Supplementary Figures 1-5. [file DataSheet_1.docx]

**Table S1. The SMRT datasets and reference genome of *Fragaria vesca* in this study**

| Cultivar | Tissue | ProjectID | Total length of subreads (Gb) | Coverage | Reference link |
| --- | --- | --- | --- | --- | --- |
| Hawaii-4 | young leaf | PRJNA383733 | 19.4 | 80.8x | https://www.ncbi.nlm.nih.gov/genome/3314 |

**Table S2. The statistical information of RNA-seq dataset in this study**

| Item | Information |
| --- | --- |
| Cultivar | Hawaii-4 |
| Tissue | leaf |
| Accession | SRR6320486 |
| Total reads num. | 8,210,530 |
| Total bases num. | 1,231,579,500 |
| Total mapped reads num. | 4,976,077 |
| Total mapped bases num. | 746,411,550 |
| Unique mapped reads num. | 4,917,135 |
| Unique mapped bases num. | 737,570,250 |

**Table S3. Results of quantitative real-time PCR**

| Group | Gene_ID | Density | 2^–ΔCt^ | Primer | |
| --- | --- | --- | --- | --- | --- |
|  |  |  |  | Forward | Reverse |
| High 6mA density | 10251608 | 0.09091 | 2.93795 | TAGAGCGGTCGGCTGTTA | CTCCCCAAGTAGGATTCG |
|  | 10251524 | 0.06977 | 1.99055 | CTCAGTGGTTAGAGTATTGC | TACCTACTATTGGATTTGAA |
|  | 10251508 | 0.03390 | 0.08127 | GACTATTGCTTTCCAG | AATTAAGGATACCCAC |
|  | 10251548 | 0.04478 | 0.29194 | ATGGAAGCATTGGTTTA | TGGTTGGAACTTTAGGC |
|  | 10251500 | 0.05634 | 4.1E-06 | TAGTTCCTACCGCTTTT | CCTTGGCTGACTGTTTT |
|  | 10251549 | 0.05479 | 2.69675 | TTTACTGGGTACGCCTTAT | TAGTCCCCGTGTTCCTC |
|  | 10251608 | 0.04571 | 3.12563 | AAAGGGTAGAAGAATGG | CTAAGAGGTGGAATAGAATA |
|  | 10251602 | 0.03929 | 5.38294 | GCCATTCGTTGGTTATT | GCTCTATTTGCCTCTGC |
|  | 10251538 | 0.02632 | 0.03370 | TGGAATTGTCTTGGGTCT | TCCAGCCAAAGTAATAGG |
|  | 10251595 | 0.03922 | 3.94187 | CGAGACGAGCCGTTTAT | CGGTCTGTTAGGATGCC |
|  | 10251601 | 0.02702 | 3.31032 | CTCAGCGGTAGAGTGTCA | ATAATCAGGCTCGAACTG |
|  | 10251607 | 0.0250 | 3.85184 | ATGGCTGAATGGTTAAAGC | GCATCCAGTAGGAATTGAA |
|  | 10251605 | 0.0250 | 1.32061 | GCCTTGGTGGTGAAAT | GAACCTCCACGCTTTT |
|  | 10251596 | 0.02379 | 1.88817 | CAACCTGGCGAACTGA | GCACGACGCTTGTATT |
|  | 10251572 | 0.02294 | 6.20024 | GCACTATCTCCCTTCAACC | CCTTCTCCGACCCTTACT |
|  | 10251571 | 0.02124 | 1.74170 | TCAGGAGGATAGATGGG | GATAAGGGATGTATGGATT |
|  | 10251570 | 0.02154 | 5.46545 | GCTTACCAAGGCGATG | TTAGCCGATGCTTATTCC |
|  | 10251576 | 0.03571 | 0.77202 | CTCAGAGGATTAGAGCAC | ACACCGTGGTTCGTAG |
|  | 10251600 | 0.01657 | 2.20379 | ATGGCTAACCCAATAAC | CACGGACAAAGTCAAGG |
|  | 10251604 | 0.01745 | 1.75849 | AAGGTAAAGCAGGGTC | CGTCATTAGTCCGATC |
|  | 10251521 | 0.0245098 | 0.805642 | CAGGAGGGATCTCTGAGG | TCTATGAAGATCGAATTCGA |
|  | 10251522 | 0.0219298 | 0.70536 | CCATGCATAAATTGAACC | TCCGTTATTATATGTTTGCT |
|  | 10251608 | 0.0457143 | 0.760913 | ATGGATGGAATCAAATATGC | AGAGTTCGACCCAATGCT |
|  | 10251585 | 0.0348259 | 0.813337 | AGCCAGTCCAATAGCTGCT | GGTATCTATGGCTTGATCAC |
|  | 10251536 | 0.02 | 0.709619 | GATCTGCTAAATTCATCGAG | GGTTATTCGTCAGCACTG |
|  | 10251530 | 0.0179641 | 0.693076 | TCGCATATTCCTGCGATT | GATAGAACTTATAGCGGGAT |
|  | 10251491 | 0.0171429 | 0.957351 | GTTCCAAACATGCCAATAT | TGATTAATGCAACTTCTAAG |
|  | 101313217 | 0.0160428 | 0.778358 | CCATCACCATGATGAAGT | CAAGCAGTGACCGTAGG |
|  | 105352753 | 0.0211268 | 2.610996 | CCTCTCTGTCCTCCATTC | ACGATTTGCAGGCGGA |
|  | 10251492 | 0.0227273 | 2.949199 | GAGCTAATGCTACAACCAGC | GATCGCGAGACAACCAGA |
| Low 6mA density | 101306897 | 7.3E-05 | 0.02917 | TGTTGAGTCATCGGGTAT | TCGCCACATTATCATTAG |
|  | 101300545 | 0.00012 | 0.00031 | AAGGAATCTGGCATCGT | GAAGGGAGTCCATCTCG |
|  | 101305618` | 7.2E-05 | 3.4E-05 | TGAGGCTGCTGATTGT | ACTCCTTCCGCATTTT |
|  | 101315323 | 9.6E-05 | 0.00020 | CCGAAAGTACAGGGACG | CTTAGAGGGCAGGTGGG |
|  | 101295796 | 0.00011 | 0.01991 | TGTATTTGCCGAAGATG | TTTGAATGATGCTGCTC |
|  | 101312616 | 0.00014 | 0.00162 | AAACGAGGTGACGAGC | CTTCAATCTAAGGGACT |
|  | 101300809 | 0.00014 | 2.1E-06 | AATGCCAAGGAGTAAC | AATCACAAGTCCCAAT |
|  | 101306941 | 0.00016 | 0.00139 | ACTATGTGATGGCTGTTACTC | CTGCTCGTCTTTGCTTGAT |
|  | 101302883 | 0.00012 | 0.00415 | AGGAACTACCTGCACTCAC | ATGCTCTGGACGCAAACTC |
|  | 101298366 | 0.00013 | 0.00113 | TCCACCATTCTTCTGCTC | CTTGCTATTTCCACTCCC |
|  | 101314193 | 0.00014 | 0.01075 | GGCAGTGAAGGATTTAG | CAACAAACCAAGGCTAT |
|  | 105350447 | 0.00011 | 0.04688 | GGCTGGATAGGAATGGTG | CTGTTTCGGTGATGGAGT |
|  | 101304521 | 0.00016 | 0.01389 | GCAGCCTACAGATGACTC | GTTAGCACCTCCATTTACT |
|  | 101306558 | 0.00013 | 0.00353 | TGTCACTCGTGCCGACCTAA | CCTAAACCAGCCATTTCCAG |
|  | 101297213 | 0.00013 | 0.00144 | TCCTCCGTTCTTCCTA | ACCGCTACTGATGCTA |
|  | 101300065 | 0.00017 | 0.05292 | TGAGTGGGTCTTTGAGTC | AGGAGGGATGAAATGTTG |
|  | 101301881 | 0.00016 | 0.01220 | TTCACCGAGCAGTCAGG | GGAATGCCAACTACTTTAT |
|  | 101314185 | 0.00018 | 3.9E-05 | CAGAACACTGCGTAAGG | CATAGCACCAACATCCT |
|  | 101291088 | 0.00017 | 0.00089 | GATACAATCTGTGAACCC | GACATTGCGGCAGTTA |
|  | 101304313 | 0.00015 | 0.00197 | TCAAGCGAGATTACATAG | AACCCAGACATAACACGAC |
|  | 101293001 | 0.000181818 | 0.016799 | TGTCATGTTTCAACAACTTG | CTGTGGACAAGGCTCTTC |
|  | 101314502 | 0.00018406 | 0.042402 | TTGGCATTAGTCATTCAGCT | CCTAGCACCATTGCTTAGA |
|  | 101306385 | 0.000190621 | 0.10239 | CTCAGTTCGTTTCTCTCTCA | GGAGTCAAGAGATGATCTTG |
|  | 101306808 | 0.000194704 | 0.011108 | AAGTTGGCAACTGCTTTG | CCCACCAAACAATCACA |
|  | 101305359 | 0.000152929 | 0.239286 | TTCTCCTGCAGGTTCATG | ATATCTTGTCAGCAAGCC |
|  | 101310409 | 0.000168379 | 0.067184 | CTGAAACAGAGAGTGAGAGA | GGTCCGAGTCCGAGTT |
|  | 105350428 | 0.000176991 | 0.270714 | CAATGGTGGGAAGGGAA | TGTAGACGACGACCTGAG |
|  | 101303746 | 0.000177211 | 0.153572 | CAGCCTTCATATTCAGGAC | CTGCACGGCTTCAGGT |
|  | 101305474 | 0.000180603 | 0.108106 | CTACTCTACCGATCAGTTACT | CTATCATCTTCCAACCTTTG |
|  | 101314743 | 0.000197902 | 0.022719 | GCTCCTAATGTGATTAGTGTG | GTATGGGAAGGTGGAAG |
| Internal control gene | LOC101296832 | \ | \ | CATTCATCACCACCGACTAC | ACCTTCTTGGCACCTCC |

ΔC_t_ = (C_t_ gene of interest - C_t_ internal control)

**Table S4. The 6mA number in lncRNA and protein-coding genes**

| LncRNA | | |  | Protein-coding genes | | |
| --- | --- | --- | --- | --- | --- | --- |
| Total no. | 6mA no. | Ratio |  | Total no. | 6mA no. | Ratio |
| 1911 | 1023 | 53.53% |  | 23106 | 15353 | 66.65% |

**Table S5. Density of 6mA across the v4.0 genomic**

| Linkage groups | Size | No.A  bases(+) | No.A  bases(-) | No. A bases | No. 6mA  sites(+) | No. 6mA  sites(-) | No. 6mA  sites | Density(-) | Density(+) | Density |
| --- | --- | --- | --- | --- | --- | --- | --- | --- | --- | --- |
| Fvb1 | 24,253,023 | 7,439,517 | 7429985 | 14,869,502 | 10,217 | 9,923 | 20,140 | 0.137% | 0.134% | 0.135% |
| Fvb2 | 29,351,230 | 8,986,528 | 8981270 | 17,967,798 | 11,185 | 11,458 | 22,643 | 0.124% | 0.128% | 0.126% |
| Fvb3 | 38,324,302 | 11,755,970 | 11772935 | 23,528,905 | 16,263 | 16,683 | 32,946 | 0.138% | 0.142% | 0.140% |
| Fvb4 | 33,907,851 | 10,363,967 | 10387674 | 20,751,641 | 15,846 | 15,641 | 31,487 | 0.153% | 0.151% | 0.152% |
| Fvb5 | 29,430,145 | 9,075,832 | 9065087 | 18,140,919 | 11,916 | 12,030 | 23,946 | 0.131% | 0.132% | 0.132% |
| Fvb6 | 39,795,230 | 12,236,789 | 12259530 | 24,496,319 | 15,790 | 15,878 | 31,668 | 0.129% | 0.130% | 0.129% |
| Fvb7 | 24,229,589 | 7,251,467 | 7301588 | 14,553,055 | 8,981 | 9,099 | 18,080 | 0.124% | 0.125% | 0.124% |
| Total | 219,291,370 | 67,110,070 | 67198069 | 134,308,139 | 90,198 | 90,712 | 180,910 | 0.937% | 0.940% | 0.135% |


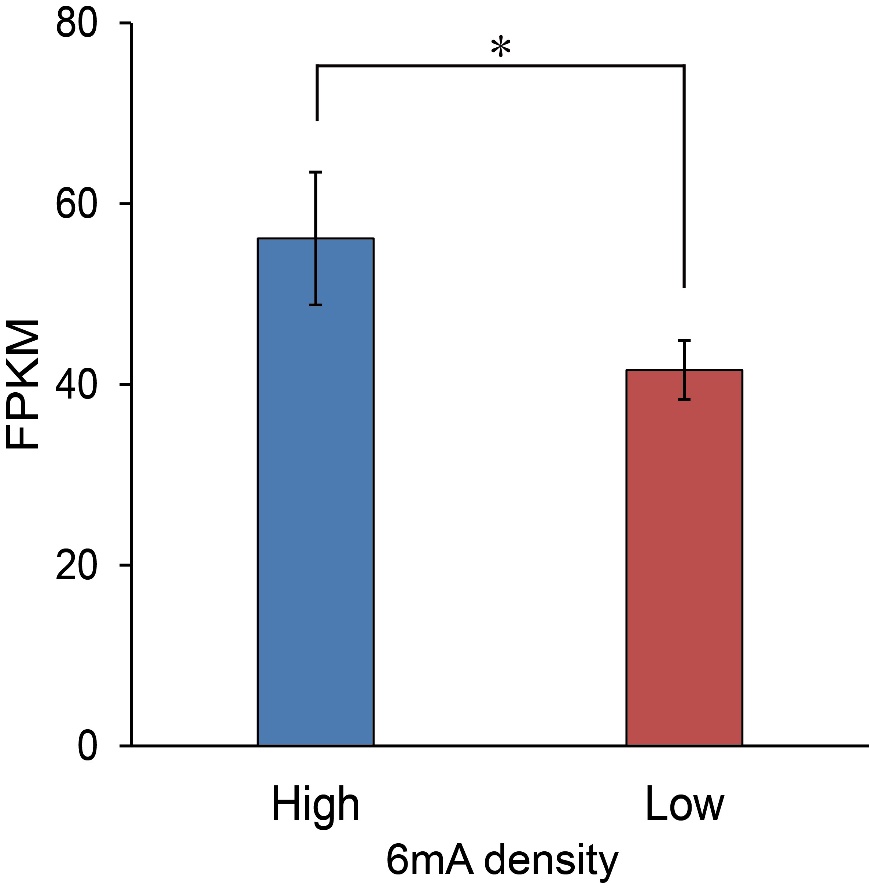


**Figure S1.** Computational analysis of the FPKM in high 6mA methylation level (n=4949) and low 6mA methylation level (n=7776) genes (mean ± SEM; *p<0.05)


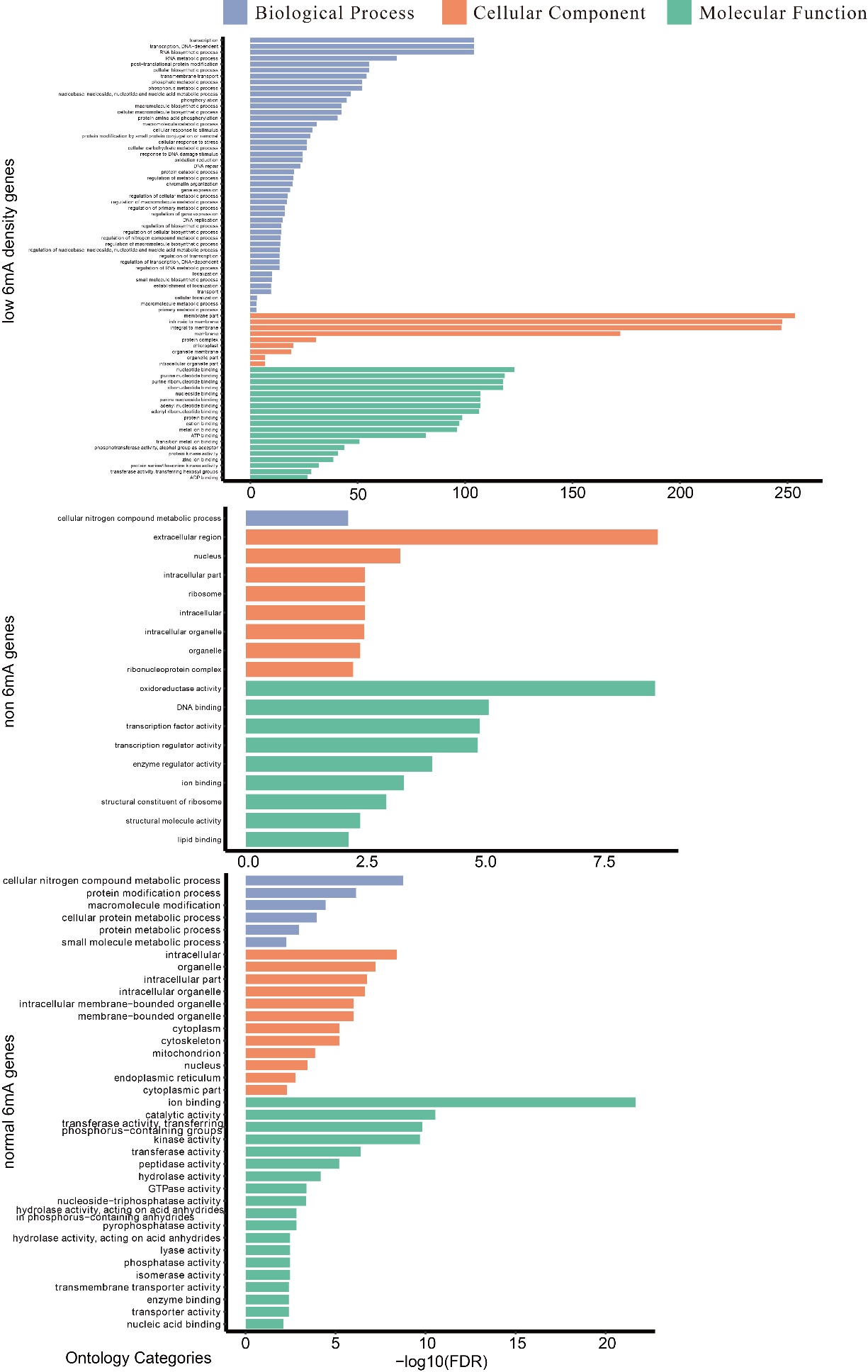


**Figure S2.** GO enrichment category of normal-6mA-level (n=10280), low-6mA-level (n=4080) and non-6mA (n=7753) protein coding genes in *Fragaria vesca*. (the GO category was listed with the adjusted P-value (FDR) < 0.01.)


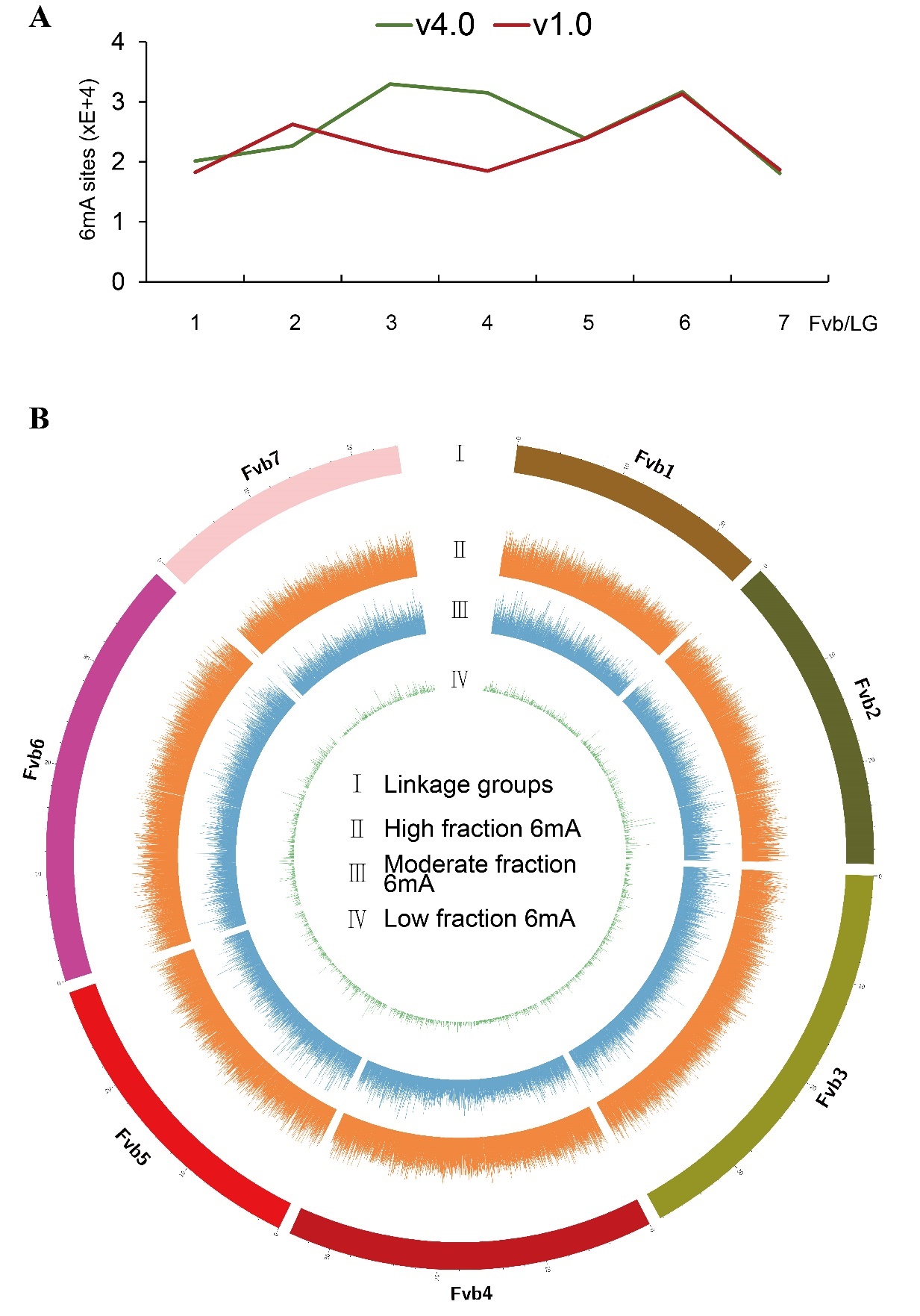


**Figure S3.** Distribution of 6mA in *Fragaria vesca* v4.0 genome. (A) Comparison of 6mA sites of seven pseudomolecules between two version genome. (B) Circos plot of 6mA in the F. vesca v4.0 genome.


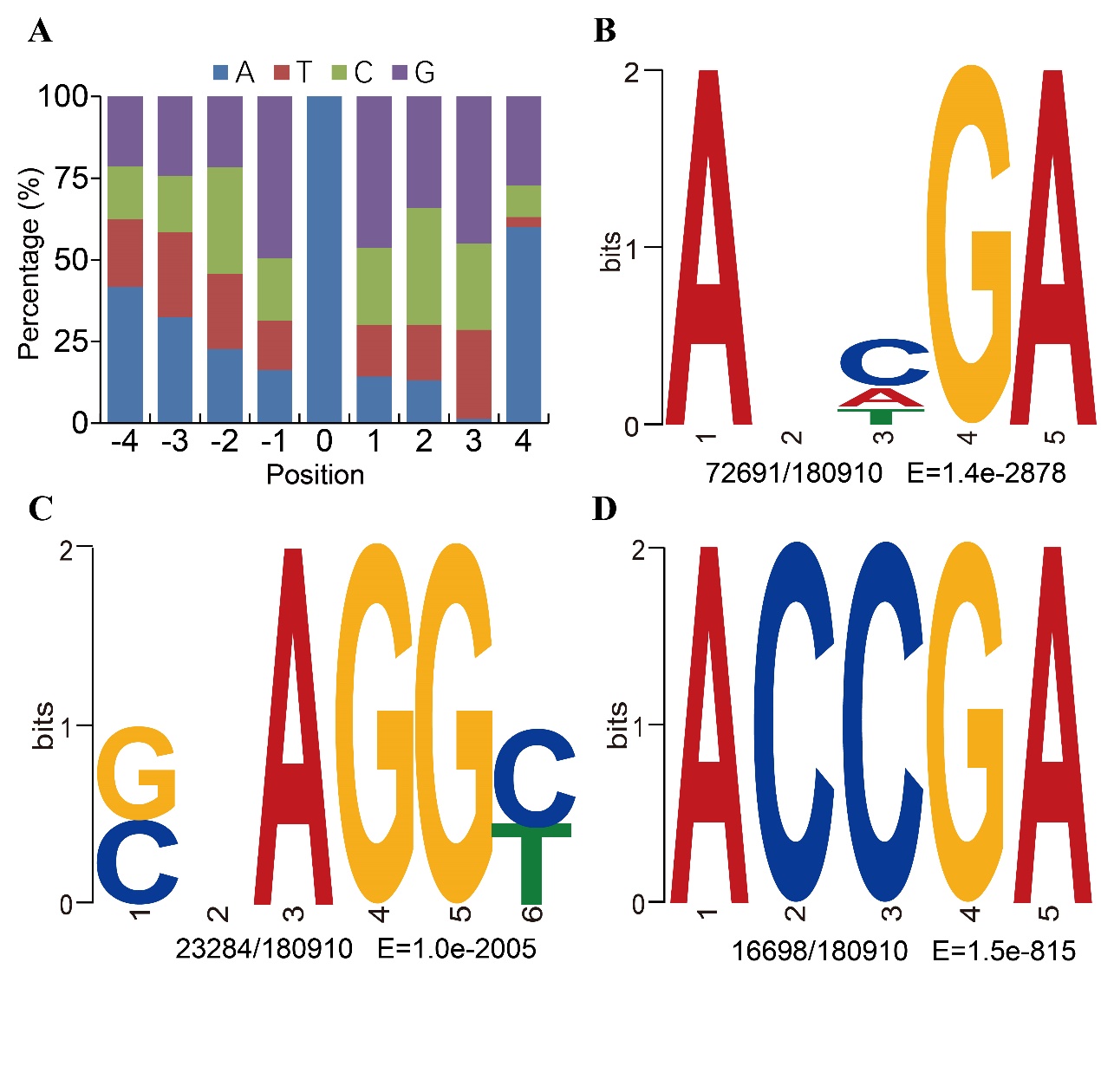


**Figure S4.** The motif sequences of 6mA sites in v4.0 genome. (A) Content percentage of the bases A, T, C, and G in the upstream and downstream 4 bp of 6mA sites (position “0”). (B-D) The motif sequences of 6mA detected by MEME assay: B, ANHGA; C, SNAGGY; D, ACCGA; Sequence logo representations of the consensus motifs containing 6mA sites identiﬁed by MEME. The number of occurrences of each motif relative to the total number of 6mA-containing motifs and the corresponding E-value generated by MEME are shown under the logo.


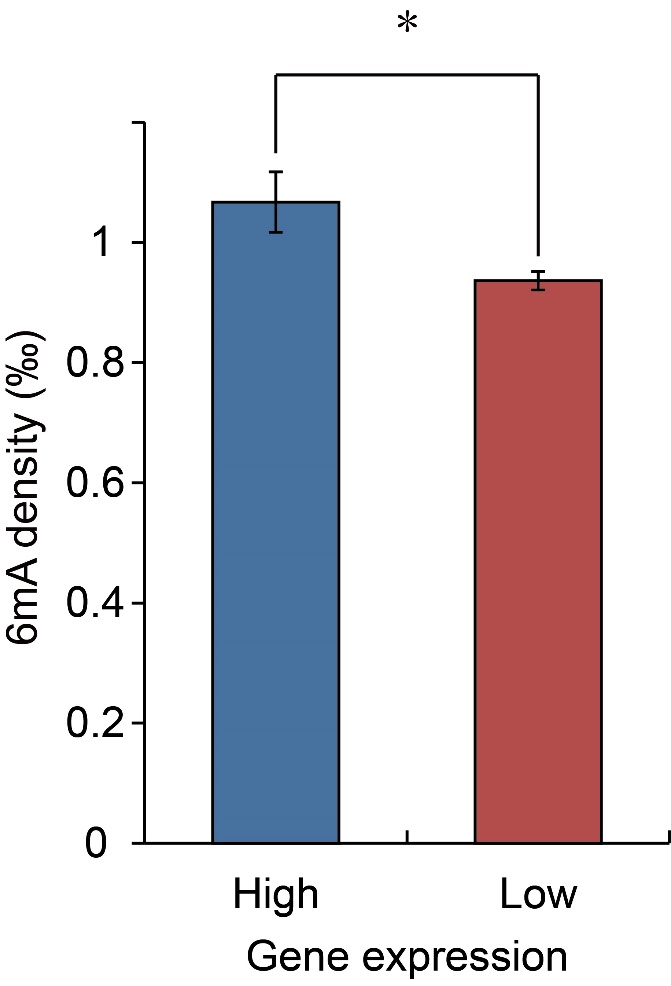


**Figure S5:** Positive association between 6mA density and gene expression using v4.0 reference genome annotation. (mean ± SEM, *p < 0.05);
